# Acceptance and commitment therapy universal prevention program for adolescents: a feasibility study

**DOI:** 10.1186/s13034-017-0164-5

**Published:** 2017-05-25

**Authors:** Rowan Burckhardt, Vijaya Manicavasagar, Philip J. Batterham, Dusan Hadzi-Pavlovic, Fiona Shand

**Affiliations:** 1 School of Psychiatry at the University of NSW, Randwick, Australia; 2 The Black Dog Institute, Hospital Rd, Randwick, NSW 2031 Australia; 3 Centre for Mental Health Research, Research School of Population Health, The Australian National University, Canberra, Australia

**Keywords:** Acceptance and commitment therapy, Adolescent, Early-intervention, Mindfulness, School, Prevention

## Abstract

**Background:**

There is a need to prevent anxiety and depression in young people and mindfulness contains important emotion regulation strategies. Acceptance and commitment therapy (ACT), a mindfulness-based therapy, has yet to be evaluated as a prevention program, but has demonstrated an ability to reduce symptoms of anxiety and depression in adult and adolescent populations. This study examines the feasibility of using an ACT-based prevention program in a sample of year 10 (aged 14–16 years) high school students from Sydney, Australia.

**Methods:**

Participants were allocated to either their usual classes or to the ACT-based intervention. Participants were followed for a period of 5 months post-intervention and completed the Flourishing Scale, Depression Anxiety Stress Scale, and a program evaluation questionnaire. Analyses were completed using intention-to-treat mixed models for repeated measures.

**Results:**

The results indicated that the intervention was acceptable to students and feasible to administer in a school setting. There were no statistically significant differences between the conditions, likely due to the small sample size (*N* = 48). However, between-group effect sizes demonstrated small to large differences for baseline to post-intervention mean scores and medium to large differences for baseline to follow-up mean scores, all favouring the ACT-based condition.

**Conclusion:**

The results suggest that an ACT-based school program has potential as a universal prevention program and merits further investigation in a larger trial.

*Trial registration* Australian New Zealand Clinical Trials Registry. Trial ID: ACTRN12616001383459. Registered 06/10/2016. Retrospectively registered.

## Background

Youth are disproportionately represented in epidemiological studies of anxiety and depressive disorders [1]. Furthermore, evidence suggests that 50% of these disorders begin before the age of 14 and 75% by age 24 years [2]. Anxiety and depressive disorders significantly impact on individuals, their families, workplaces, communities, and countries [3–5], making prevention an important goal. Compared to treatment in the early stages of the disorder, termed ‘early intervention’, prevention averts the short- and long-term consequences of such disorders and has been shown to be more cost-effective [6, 7]. Prevention programs that are delivered to all individuals irrespective of their level of symptomatology, termed ‘universal prevention’, reduce the logistical difficulties involved in large-scale screening, avert missing susceptible students, and ensure that the benefits of such a program are available to all. Existing prevention programs have largely drawn these skills from cognitive behavioural therapy (CBT), which focuses primarily on teaching individuals to change their appraisal of a situation in order to modify the emotional experience [8–11].

Mindfulness, a particular manner of engaging with one’s environment, is a concept that has grown in popularity over recent years. It has been defined as comprising two components—paying attention to the present moment and doing so with a non-judgmental attitude [12]. These two components correspond closely with two well-established emotion regulation strategies: attention deployment and acceptance [13, 14]. Attention deployment is the ability to choose which aspect of a situation to focus on while acceptance involves allowing an emotion to occur without attempting to avoid the experience. With emotion regulation implicated in the development of mental disorders, particularly to anxiety disorders and depression [14–17], it is unsurprising that numerous clinical trials have demonstrated that mindfulness can assist with a wide range of mental health symptoms in adult populations [18–28].

There are reasons to believe that mindfulness may be beneficial to adolescents in navigating their environment. Developmental brain changes mean that adolescents are more impacted by emotions than adults or children: they have increased limbic reactivity (indicative of a greater sensitivity to threat), a stronger startle reflex (suggesting a more intense automatic emotional reactivity), and are more interfered by emotional stimuli when completing tasks [29–32]. In spite of adolescents experiencing stronger emotional reactions, the frontal lobe of the brain, which controls the executive functions such as judgment, impulse control, planning, and emotion regulation, remains underdeveloped [33]. Mindfulness and its ability to regulate emotions, may help counteract this imbalance. Research has demonstrated that mindfulness can reduce mental health symptoms in both clinical and non-clinical adolescent populations [34].

There are a number of existing programs that have been designed to train individuals to become more mindful such as mindfulness-based stress reduction (MBSR), mindfulness-based cognitive therapy (MBCT), and acceptance and commitment therapy (ACT). MBSR has a focus on teaching meditation practices, which serve to train the brain to become more mindful (similar to using weight training to increase physical strength). MBCT combines meditational practices with components of cognitive-behavioural therapy for depression. Unlike MBCT and MBSR, ACT does not use meditation but rather is didactic in style and draws heavily on imagery, metaphors, personal stories, and short experiential exercises. It also focuses on the application of mindfulness to emotions and related internal constructs such as thoughts, memories, and body sensations. In addition to mindfulness, ACT draws on the concept of ‘values’ as an over-arching framework to guide the intervention techniques, improve life-satisfaction, and increase motivation. ACT defines values as the type of person an individual wishes to be in the future. Behavioural principles are also incorporated into ACT to assist a person in working towards their values. Engaging in such behaviours that are important and goal-directed even while experiencing intense emotions, may be regarded as a form of emotion regulation [35]. Acceptance principles are encouraged when thoughts and feelings draw the individual away from maintaining value consistent behaviours. ACT does not distinguish between psychopathology and everyday struggles and so, is equally applicable to those with or without significant psychopathology. While ACT was developed for adults, it has been successfully applied to adolescent clinical populations [36–38]. When used with adolescents, ACT principals remain the same but exercises, examples, and metaphors employed will be more age appropriate [39].

Acceptance and commitment therapy is an appealing mindfulness program to use with adolescents compared to other meditation-based programs because adolescents may struggle to engage with meditation, particularly in a school setting. It is also appealing for use with adolescents as it places particular emphasis on using mindfulness to regulate emotions, which is important for this age group. Finally, the additional components of ACT such as values may be particularly useful for adolescents as they are in an important transitional period where they are creating a self-identity. ACT has been shown to be effective in clinical samples of adolescents to address symptoms of depression and anxiety [37, 38] but remains untested as a prevention program (i.e. in a non-clinical sample of adolescents). The results from clinical samples suggest that ACT is effective as an emotion regulation strategy and so is worth evaluating as a prevention program. There are nonetheless differences in using ACT as a therapy compared to as a prevention program. Many ACT-based therapies will incorporate other elements to target the disorder (e.g. behavioural activation for depression or exposure for anxiety) whereas in prevention this may be less relevant. Instead it may be more important to emphasise how experiential avoidance can lead to many types of problems and techniques that can be used to reduce experiential avoidance. Furthermore, the examples used to illustrate how ACT can be applied to real-life situations in clinical therapies may need to be modified to make ACT more relevant to a non-clinical population.

The present study sought to investigate for the first time an ACT-based intervention as a school universal prevention program. One problem in the evaluation of a new prevention program is the issue of statistical power. Given there is a low rate of emergence of new cases of anxiety and depression over any given period, it has been estimated that over 30,000 participants are needed to adequately power a prevention program evaluation study [40]. Measuring symptom reduction rather than cases can reduce this figure but still almost 1000 would be required to demonstrate a statistically significant difference between conditions [41]. However to justify investment in a trial of this size, preliminary evidence, obtained in a feasibility study, is required. The aims of this study were to: (a) examine the feasibility and acceptability of using an ACT-based prevention program that targets anxiety and depressive symptoms in a non-clinical sample of adolescents; and (b) to compare the impact of the ACT-based program on wellbeing and symptoms of depression and anxiety. It was expected that there would be a trend for the ACT participants to demonstrate improvements on a range of measures compared to participants in the control condition. Given the underpowered nature of feasibility studies, this trial sought to use effect sizes as an indication of feasibility and to provide evidence to determine whether a large-scale prevention study of this intervention would be appropriate.

## Methods

### Participants

**Fig. 1:**
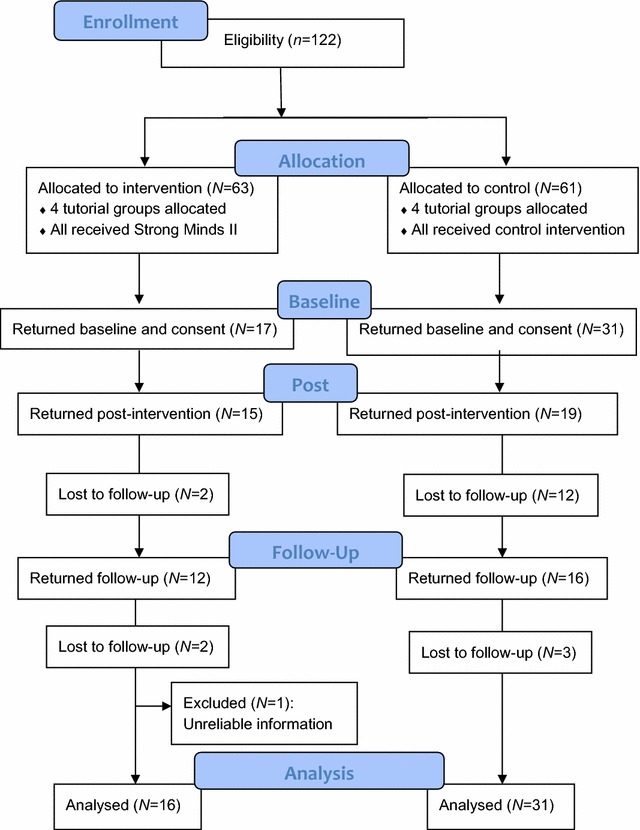
Flow chart of participants’ progression through the study

Participants were drawn from a private high school located in Sydney, Australia. Students attending this school are socio-economically advantaged compared with other students in the state of New South Wales and Australia, with 76% in the top quartile on a measure of socio-educational advantage [42]. The school performs well academically and is ranked in the top 15% of schools in New South Wales [43]. All students in year 10 (*N* = 122) were invited to participate in the study and, if they agreed, were required to provide signed parental and self-consent. Regardless of participation in the evaluation study, all year 10 students attended the ACT workshops as it was required curriculum. Year 10 in the Australian school system is the 3rd year completed before graduating from high school. Ethical approval was obtained from the University of New South Wales Human Research Ethics Committee (reference HC13132). Of the 122 students in year 10 who were invited to take part in the study, 76 were males (62%). Forty-nine participants provided self- and parental-consent. The primary reason the remaining students did not take part was due to not returning signed parental consent forms. Of the 49 participants who provided self- and parental-consent, one student’s data had to be discarded because of unreliable information. A flow chart presents participants progression through the study (Fig. 1). The final sample comprised 48 participants aged between 14 and 16 years (*M* = 15.64), of whom 28 were male (58%).

### Interventions

#### ACT condition

An ACT-based prevention program was developed by two of the authors (RB and VM) that utilises the ACT components of: values, committed action, contact with present moment, acceptance of emotions, and thought defusion. These components are included in other ACT trials and are consistent with the manner in which ACT is described by the original authors of ACT [44]. While ACT traditionally includes a section on the component of self-as-context, the technique of detaching from the experience, this was dropped from the current intervention as the facilitator (RB) has found that it is a difficult and confusing concept to transmit to adolescents. Many programs make changes to the original therapy in order to better suit the population targeted [e.g. 36, 45] and other ACT interventions have also chosen not to include this component [46]. The lead author delivered each session of the ACT program face-to-face to half of year 10 (approximately 60 students, 30% of whom were enrolled in the study) in an amphitheatre at the school. The aim of each session was to educate the students on a particular concept (e.g. values) and encourage them to apply the concepts to their everyday life. To achieve this aim in each session, verbal explanations, personal stories, metaphors, PowerPoint slides, videos, and experiential exercises were used. An example of an experiential exercise was the mindful eating of a sultana to help teach the concept of contact with present moment. The lecture-style presentations were supported by four teacher-led exercises between the presentations, during class time and in smaller groups (approximately 15 students). Teacher exercises, outlined in Table 1, were based on the ACT components of thought defusion, contact with present moment, and values. All workshops and teacher-led classes were 25 min in duration. In total, students received 4.6 h of the intervention.

**Table 1:** Overview of the ACT program

Exercise	ACT component	Exercise
1	Values	Debate between students on whether it is better to follow values-based living or feeling good
2	Values	Completing a booklet that guides students through a series of questions to help them identify their values
3	Thought defusion	Acting in a short play where characters’ thoughts are spoken out loud
4	Contact with present moment	Students choose and listen to several mindfulness audio tracks from a pool of various tracks

#### The control condition

The control condition comprised ‘Pastoral Care’, which was their usual class activity during the time slot that the ACT workshops were being delivered. In these classes of around 15 students, material on social justice and cyber-safety was presented by a schoolteacher who had been assigned to the tutorial at the start of the semester, prior to the study commencing. Duration of both programs was equivalent—all Pastoral Care classes were 25 min in duration and students received 4.6 h of Pastoral Care.

### Procedure

#### Recruitment and procedure

The program was delivered to half year 10 students in Term 1 and half in Term 3. The school made attending the workshops obligatory but participation in the study was voluntary. Prior to the first session, participants filled out baseline questionnaires and students who were absent on the day were approached up to 1 week later. Workshops began 1 week after the baseline questionnaires were completed and were delivered once per week for 7 weeks. At the end of the last ACT workshop, participants completed a program evaluation survey. The post-intervention questionnaire was administered 1 week after the final workshop. Attempts were made to contact absent students for a period of up to 2 weeks. The follow-up questionnaire was administered 5 months after the baseline questionnaire was completed. After the final follow-up questionnaires were collected, the ACT program was delivered to the control condition.

#### Design and randomisation

The current study was a quasi-randomised controlled study. Randomisation was conducted by a staff member of the school who was independent of the study using cluster randomisation based on their tutorial groups of which there were eight in the year group. Tutorial groups had a name and these were listed alphabetically and the first assigned to the ACT condition and the last four to the control condition. Randomisation was completed before enrolment in the study because attendance to the workshops was a required curricular activity for students. It was for this reason that there were disproportionate numbers in each condition and the randomisation was considered ‘quasi’.

#### The ACT program facilitator

The ACT workshop facilitator (RB) had 6 years of university training in psychology, including 2 years in a clinical master’s program. He had also received specialist training and supervision in ACT and had previously delivered the same material to a similar sized group of students in the same school. At the time of workshop delivery, he had approximately 2 years’ experience using ACT in both an individual and group context.

### Measures

A questionnaire for anxiety and depression symptoms and a wellbeing scale were selected as outcome measures. While it was acknowledged that there are inherent difficulties in using a measure of anxiety and depression created for clinical populations in a non-clinical sample, it was esteemed that it was the best choice of the alternatives available. The symptom measure selected has been used and validated in a non-clinical adolescent population [47–49]. In addition, a program evaluation questionnaire was created specifically for this study. Finally, while a fidelity scale created for another study [50] was used, technical problems meant the data was not obtained.

#### The Depression Anxiety and Stress Scale—Short Form (DASS-21)

The DASS-21 is a widely used measure of negative affect [51]. It comprises 21 items scored on a 4-point Likert scale. For adults, items load onto three subscales—depressive symptoms, anxiety symptoms, and stress symptoms. Both the anxiety and stress subscale are measures of anxiety, the former are symptoms found in phobias while the latter is consistent with generalized anxiety disorder. For adolescents some authors found the same loading structure as for adults whereas others found a single loading—negative affect [47–49]. Given these discrepancies, both the three subscales and a single total score were used in the current study. For adolescents, the Cronbach’s α has been found to be .87 for the depression subscale, .79 for the anxiety subscale, and .83 for the stress subscale [48]. In the current study, the Cronbach’s α was .90 for the depression subscale, .81 for anxiety, .88 for stress, and .95 for DASS-Total.

#### Flourishing Scale (FS)

The FS is a 7-item measure of wellbeing with responses given on a 7-point Likert scale [52]. Items assess several domains of wellbeing including social relationships, competency in activities, optimism, purpose and meaning, and self-esteem. The FS has a single factor structure with item loadings between .72 and .81 [53]. The scale also correlates negatively with the Centre for Epidemiologic Studies—Depression Scale (−.60). Amongst the 18–20 year-old cohort in this study, the mean score was 42.71 (*SD* = 7.96) with a Cronbach’s α of .87.

#### Program evaluation

A series of 10 questions with a 6-point Likert scale was created for this study: “Because of the workshops… (a) I’m clearer about what’s important to me in life (values); (b) I am more comfortable sitting with negative emotions; (c) My negative thoughts impact me less; d) I am exercising more; (e) I feel happier; (f) I feel more confident; (g) Anxiety is less of a problem for me; (h) I am getting along better with friends or family; (i) I have found that the workshops/tutorials have helped me; (j) I have been applying what I learnt in the workshops/tutorials to my everyday life.” The measure was delivered at the post-intervention time-point only.

### Statistical analysis

Statistical analysis was completed using SPSS 22.0. For the DASS-21 subscales, at least six of the seven items were required to compute a mean and seven of the eight items for the FS, as per author guidelines. For baseline and dropout comparisons, *t*-tests were used for continuous variables and Chi square tests of independence with Yates Continuity Correction for categorical variables. In the universal effects analysis comparing outcome scores between the ACT and control conditions an intention-to-treat analysis was used. Mixed models with repeated measures (MMRM) is considered to be particularly appropriate for school-based studies or when the dropout rate is above 5% as it includes all available data from both completers and dropouts [54]. A single analysis examined the outcome scores across the three time-points. To select the covariance structure, the best fitting model using Akaike’s information criterion was retained. The parameters of the model were estimated using restricted maximum likelihood as the evidence suggests it is preferable over maximum likelihood when the sample is small [55]. The *d*_ppc2,_ formula [56] was used to estimate Cohen’s *d* effect sizes, except for the Chi square tests that Phi (*φ*) effect size, both of which were interpreted using Cohen’s standards [57]. A positive effect size on the DASS-21 and FS scales represent improvement in symptoms and wellbeing, respectively.

## Results

Mean scores and standard deviations for the ACT and control condition are presented in Table 2.

**Table 2:** Means (*SDs*) for mental health measures at each time point, *N* = 48

Measure	Condition	Baseline	Post	Follow-up
DASS-depression	ACT	7.34 (7.85), *n* = 17	7.38 (6.81), *n* = 15	6.00 (5.59), *n* = 12
	Control	9.27 (10.95), *n* = 31	12.33 (12.25), *n* = 19	11.25 (10.75), *n* = 16
DASS-anxiety	ACT	9.06 (7.22), *n* = 17	8.00 (6.55), *n* = 15	5.33 (4.03), *n* = 12
	Control	9.42 (8.47), *n* = 31	10.63 (9.59), *n* = 19	10.19 (10.08), *n* = 16
DASS-stress	ACT	13.29 (7.87), *n* = 17	11.07 (6.32), *n* = 15	9.00 (5.62), 12
	Control	11.48 (8.75), *n* = 31	14.70 (11.17), *n* = 19	13.63 (9.75), *n* = 16
DASS-total	ACT	29.78 (20.99), *n* = 17	26.44 (16.75), *n* = 15	20.33 (12.26), *n* = 12
	Control	30.17 (25.57), *n* = 31	37.67 (29.72), *n* = 19	35.06 (28.48), *n* = 16
FS	ACT	46.47 (7.19), *n* = 17	46.64 (9.47), *n* = 14	45.64 (7.12), *n* = 11
	Control	44.45 (7.40), *n* = 31	43.11 (9.80), *n* = 19	41.30 (8.36), *n* = 15

### Baseline comparisons

The comparison of baseline scores of the ACT and control conditions found no significant differences for DASS-depression (*p* = .55), DASS-anxiety (*p* = .88), DASS-stress (*p* = .48), DASS-total (*p* = .96), or FS (*p* = .37). No significant difference in sex distribution was found between the conditions (*p* = .72).

### Comparison of dropouts

Comparisons of the differences between participants that completed their post-intervention and follow-up questionnaires (‘completers’) from those who did not (‘dropouts’) suggested there were no significant differences between post-intervention completers and dropouts on DASS-depression (*p* = .69, Cohen’s *d* = .14), DASS-anxiety (*p* = .88, Cohen’s *d* = .05), DASS-stress (*p* = .72, Cohen’s *d* = .11), DASS-total (*p* = .81, Cohen’s *d* = .08), and FS (*p* = .93, Cohen’s *d* = .02). Likewise, no differences were found between follow-up dropouts and completers scores for DASS-depression (*p* = .36, Cohen’s *d* = .27), DASS-anxiety (*p* = .18, Cohen’s *d* = .41), DASS-stress (*p* = .29, Cohen’s *d* = .33), DASS-total (*p* = .24, Cohen’s *d* = .36), and FS (*p* = .28, Cohen’s *d* = .32). No significant differences were detected on the variable sex between the completers and dropouts at post-intervention (*p* = .67, *φ* = .11) or follow-up (*p* = .92, *φ* = .06). Likewise, no differences in attrition were observed between the ACT and control conditions at post-intervention (*p* = .10, *φ* = .27) or follow-up (*p* = .20, *φ* = .18).

### Universal effects of the program

The MMRM findings from the Time × Condition analyses are presented in Table 3. There were no significant differences between the ACT and control conditions for the DASS-21 or FS. However, given this was a pilot study in a non-clinical sample, we were chiefly interested in whether effect sizes suggested that a larger trial of the intervention would be worthwhile. Effect sizes were calculated for both baseline-post and baseline-follow-up differences. Table 4 presents Cohen’s *d* effect sizes observed for the difference between the ACT and control conditions. Results indicate medium or large between-group effect sizes for all outcomes for baseline to follow-up differences.

**Table 3:** Linear mixed modelling time × condition results, type III fixed effects, *N* = 48

Measure	DF	*F*	*P*
DASS-depression	1.61	.24	.79
DASS-anxiety	1.60	1.72	.19
DASS-stress	1.61	2.05	.14
DASS-total	1.60	1.39	.26
FS (Wellbeing)	1.45	.57	.57

**Table 4:** Cohen’s *d* effect sizes and standard interpretations, *N* = 48

Measure	Baseline-post	Cohen’s standard	Baseline-follow-up	Cohen’s standard
DASS-depression	.31	Small	.34	Medium
DASS-anxiety	.28	Small	.55	Medium
DASS-stress	.63	Large	.75	Large
DASS-total	.44	Medium	.59	Medium
FS	.20	Small	.31	Medium

### Program evaluation

Twenty-four participants, from both conditions, completed the ACT program evaluation. Program evaluation data are summarized in Table 5. To assess the percentage of affirmative/negative responses, the ‘Strongly Disagree’, ‘Moderately Disagree’, and ‘Slightly Disagree’ responses were combined into a single outcome ‘Disagree’ and the same done for the three agree options which were combined into ‘Agree’. Overall, there was agreement with the questions posed, except in response to whether they were exercising more.

**Table 5:** Means and percentage of participants agreeing on the program evaluation questions, *N* = 24

Question	*n*	Mean	SD	% Agreeing
More confident	24	4.1	1.4	75
Less impact of negative thoughts	23	4.0	1.2	70
Happier	24	4.0	1.7	67
Workshops helpful	24	3.8	1.4	63
More comfortable with negative emotions	24	3.8	1.1	63
Values clearer	24	3.6	1.6	58
Better relationships	24	3.5	1.8	54
Less impacted by anxiety	24	3.5	1.7	50
Applying workshops to everyday life	24	3.6	1.2	50
Exercising more	24	2.5	1.7	29

## Discussion

The current study investigated the feasibility of using an ACT-based prevention program for adolescents in a school setting. As expected because of the small sample size, the analyses indicated that there were no statistically significant differences between the ACT and control conditions on the outcome measures of depression, stress, anxiety, total negative affect, and wellbeing. The effect sizes, which were expected to be of greater utility in this study, ranged from small to large according to Cohen’s standards [57], all in the direction of greater improvements in the ACT compared to control condition. The high rate of endorsement of the various items in the workshop evaluation questionnaire also suggests that many participants perceived benefits from the workshops. To our knowledge, this is the first time that an ACT-based program has been evaluated as a prevention program in a non-clinical population of adolescents. It is conceivable that with a larger sample size, a universal prevention evaluation study of an ACT-based program may find mean differences that are statistically significant.

Interestingly, the present study found that stress scores demonstrated greater improvements over time than anxiety scores on the DASS-21. While both are broad measures of anxiety, the stress scale of the DASS-21 relates to cognitive symptoms (i.e. worry) while the anxiety subscale tends to relate to physiological symptoms (e.g. increased heart beat). Given that worry may be regarded as a form of avoidance [58], it is unsurprising that this aspect would be more amendable to change for participants in the ACT intervention compared to physical symptoms that are not associated with avoidance behaviours.

Compared to the findings from CBT prevention programs, our results are encouraging. A meta-analysis of prevention programs for depression [10] found that the average effect size from baseline to post-intervention was Pearson’s *r* = .15 (Cohen’s *d* = .30), which is equivalent to our results. Effect sizes at follow-up were Pearson’s *r* = .11 (Cohen’s *d* = .20), which is lower than the effect size found in the present study. Early intervention programs targeting depression and other mental health-related problems have reported a reduction in efficacy from post-intervention to follow-up [10, 59]. These programs were predominately CBT-based and thus focused on teaching skills, the retention of which is likely to diminish over time. The present intervention, on the other hand, observed an increase in efficacy from post-intervention to follow-up. It is possible that ACT may create a more fundamental shift in how a person relates to their thoughts and feelings rather than teaching a new set of skills [60].

The program evaluation questions showed strong satisfaction with the ACT program. The item which most students agreed with related to increased confidence from the workshops. This is likely to reflect that many young people struggle with low confidence. Despite two-thirds of participants reporting that the workshops were helpful, three-quarters said they felt more confident because of them, which may be reflective of a tendency amongst adolescents to downplay positive changes.

The present study used a large group setting to deliver the workshops, with approximately 60 students attending the workshop at the same time. The findings suggest that future early intervention programs may not need to be delivered to small groups. Large groups enable programs to be more readily delivered by external psychologists (due to reduced costs) rather than teachers which is an important advantage given that research suggests that compared to teacher-led programs, psychologist facilitated programs have better outcomes [59, 61]. In addition, it was observed that in the present study, students asked clarifying questions on the workshop material that schoolteachers without psychology training/experience would have difficulty in answering. The responses to these questions appeared important to ensure students understood the material taught. For these reasons, we recommend that trained psychologists rather than schoolteachers deliver the program.

This study did have a number of methodological issues that limit the conclusions that can be drawn, including the small sample size and the quasi-randomisation process. Although the randomisation method led to unequal numbers of participants in each condition, importantly, baseline differences between conditions were not statistically different. Another limitation was the inability to differentiate the benefits obtained from the practical teacher-led exercises compared to the psychologist-led workshops. Future research to examine their differential benefits would be of interest. The study was also limited in that the sample used was a private school with students from high socio-economic status families. It would be of interest to evaluate the program across the socio-economic spectrum. The present study experienced a substantial dropout of participants in the control condition between baseline and post-intervention. The workshops ended close to school holidays and so the limited time available before students left meant that the students in the control condition, absent on the day the questionnaires were administered, could not be located. This learning can be utilised in future school-based program evaluation research to avoid this same issue.

There are a number of components that would be of benefit to include in a larger trial of an ACT-based prevention program. Examining an increased range of outcomes, such as academic indicators and social relationships (e.g. family, friendships, teacher–student) in addition to the mental health outcomes, would be of interest. The study design could also have been improved by including a measure of emotion regulation, and it is recommended that future studies in this area do so. In addition, it would be of benefit that such a trial test the model that mindfulness reduces and prevents depressive and anxiety symptoms through improved emotion regulation. Including mediator variables and testing this model through analysis would provide greater insight into the link between mindfulness and symptom reduction/prevention. Future trials could also be strengthened by examining potential confounds and moderators of outcome, such as previous experience with mindfulness, current engagement with mental health professionals, student tutorial group, school exams during the study period, and past engagement with mental health prevention workshops. Finally, although it would be resource intensive, a future trial that can compare the effectiveness of several key emotion regulation strategies (e.g. problem-solving, cognitive re-appraisals, and acceptance) as well as a combined program of all these together, would be of great benefit to the field of prevention.

## Conclusions

The results from the current feasibility study found that an ACT-based prevention program delivered in a school setting led to moderate to large effect size differences between the conditions at the 5 month follow-up and that the program was feasible and acceptable to participants. This study suggests that an ACT-based program should be examined further in a larger and more representative sample. A number of lessons can be drawn from this study to inform such a trial.

## Abbreviations

ACT: acceptance and commitment therapy
CBT: cognitive behavioural therapy
DASS-21: The Depression Anxiety and Stress Scale—Short Form
MBCT: mindfulness-based cognitive therapy
MBSR: mindfulness-based stress reduction
MMRM: mixed models with repeated measures
FS: Flourishing Scale
